# Cripto-1 overexpression is involved in the tumorigenesis of nasopharyngeal carcinoma

**DOI:** 10.1186/1471-2407-9-315

**Published:** 2009-09-06

**Authors:** Zhengrong Wu, Gang Li, Lirong Wu, Desheng Weng, Xiangping Li, Kaitai Yao

**Affiliations:** 1 Department of Pathology & Guangdong Provincial Key Laboratory of Molecular Tumor Pathology, School of Basic Medical Sciences, Southern Medical University, Guangzhou 510515, PR China; 2 Department of Otorhinolaryngology Head and Neck Surgery, Nanfang Hospital, Southern Medical University, Guangzhou 510515, PR China; 3 College of Traditional Chinese Medicine, Guangzhou University of Chinese Medicine, Guangzhou 510006, PR China; 4 Department of Medicine, Boston University School of Medicine, Boston, MA 02118, USA; 5 Cancer Research Institute, Southern Medical University, Guangzhou 510515, PR China

## Abstract

**Background:**

Human Cripto-1, a member of the EGF-CFC family, is indispensable for early embryonic development. Cripto-1 plays an important oncogenic role during tumorigenesis and is overexpressed in a wide range of epithelial carcinomas, yet little is known about Cripto-1 in nasopharyngeal carcinoma (NPC). The aim of this study was to analyze the roles of Cripto-1 in the progression and clinical characteristics in NPC clinical samples and cell lines.

**Methods:**

The expression of Cripto-1 at mRNA level was detected by the reverse transcription-polymerase chain reaction (RT-PCR) and real time RT-PCR, and western blot was used to examine the protein expression. Cripto-1 expression and its clinical characteristics were investigated by performing immunohistochemical analysis on a total of 37 NPC clinical tissue samples. Lentiviral vectors were constructed to get an efficient expression of anti-Cripto-1 siRNA in CNE-2 and C666-1 cells, with invalid RNAi sequence as control. After the inhibition of the endogenous Cripto-1, the growth, cell cycle and invasion of cells were detected by MTT, FACS and Boyden chamber assay respectively. Moreover, *in vivo*, the proliferation of the tumor cells was evaluated in xenotransplant nude mice model with whole-body visualizing instrument.

**Results:**

The results of real-time RT-PCR and western blot showed that the expression level of Cripto-1 was markedly higher in NPC cell lines than that in the immortalized nasopharyngeal epithelial cell at both mRNA and protein levels. RT-PCR of 17 NPC tissues showed a high expression rate in 76.5% (13/17) cases. In an immunohistochemical study, Cripto-1 was found to express in 54.1% (20/37) cases of NPC. In addition, Cripto-1 overexpression was significantly associated with N classification (*p *= 0.034), distant metastasis (*p *= 0.036), and clinical stage (*p = *0.007). Inhibition of endogenous Cripto-1 by lentivirus-mediated RNAi silencing technique suppressed NPC cell growth and invasion *in vitro*. *In vivo*, the average weight (*p *= 0.026) and volume (*p *= 0.044) of tumor in CNE-2/GFP^+^/Cripto-1^- ^xenotransplant mice group were significantly lower than those in the control group. The Ki67 index was obviously lower in Cripto-1 RNAi treated tumors (*p *< 0.01).

**Conclusion:**

Data of this study suggest that Cripto-1 overexpression is connected with the tumorigenesis and progression of NPC, lentivector-mediated RNAi might be feasible for the inhibition of the growth and invasion of NPC.

## Background

Nasopharyngeal carcinoma (NPC) is a distinctive type of head and neck cancer with special racial and geographic distributions. It is one of the most common cancers in Southeast Asia and Southern China. The incidence rate of NPC in Southern China (20-50/100,000 people per year) is nearly 100-fold higher than that in the western world[1]. Latent Epstein-Barr virus (EBV) infection is uniquely present in almost all NPC from endemic regions, whereas absent in NPC from nonendemic regions[2,3]. Additionally, NPC originates from a hidden anatomical site, and is more closely associated with advanced clinical stage with higher incidence of invasion and metastasis at the time of diagnosis. Hence, the prognosis for NPC patients is poor with a 5-year survival rate of less than 60%. It is of great clinical value to further understand the molecular mechanism of this cancer and find valuable early diagnostic markers as well as novel therapeutic strategies.

Human Cripto-1, also known as teratocarcinoma-derived growth factor-1 (TDGF-1), is a member of the *Epidermal growth factor-cripto FRL1 cryptic *(EGF-CFC) family (Cripto in humans, FRL1 in Xenopus, and Cryptic in mice), which is indispensable for early embryonic development[4]. *In vivo*, this 188-amino acid glycoprotein, Cripto-1 has two activity patterns: as a cell surface co-receptor anchored by glycosylphosphatidylinositol and as a soluble protein after cleavage of the glycosylphosphatidylinositol linkage[5]. Although identified as a marker for embryonic stem cells and generally absent from adult tissues, Cripto-1 is overexpressed in 75-80% of human breast, colon, and lung cancers, as well as 50-60% of testicular, stomach, pancreatic, and ovarian cancers[6]. Furthermore, Cripto-1 expression is significantly increased in premalignant lesions, such as colon adenomas, intestinal metaplasia of the gastric mucosa and ductal carcinoma in situ (DCIS) of the breast[7]. Recently, it has been reported that plasma Cripto-1 might represent a novel biomarker for the early detection of breast and colon carcinomas[8]. In an additional study, combined analysis of Cripto-1 and E-cadherin has significant value in evaluating the metastatic potential of gastric cancer and predicting patient prognosis[6]. *In vitro *and transgenic mice studies have shown that cripto-1 play an important oncogenic role during tumorigenesis by promoting cell proliferation, survival, migration and invasion, as well inducing epithelial-to-mesenchymal transition(EMT), transformation, branching morphogenesis and tumour angiogenesis[9,10].

Until now, there is never any evidence that has shown a relationship between Cripto-1 expression and carcinogenesis of NPC. In this investigation, we examined the Cripto-1 expression in human NPC in both clinical patient samples and tumour cell lines. We also used small hairpin RNA (shRNA) technique based on lentivirus vector to specifically inhibit the expression of Cripto-1 in a NPC cell line CNE-2. Our results provide strong evidences that Cripto-1 is upregulated in human NPC and might play a role in malignant progression of NPC.

## Methods

### Cell lines

The human NPC cell lines C666-1, CNE-1, CNE-2, HNE-1, SUNE-1, and HONE-1 were grown in RPMI-1640 medium (Hyclone, Logan, UT) supplemented with 10% fetal calf serum (ExCell, Shanghai, China) and 1% L-glutamine. NP69, a human immortalized nasopharyngeal epithelial cell line[11], was grown in defined-KSFM medium supplemented with EGF (Invitrogen, Carlsbad, CA). Human embryonic kidney cell line 293FT was grown in DMEM supplemented with 10% fetal calf serum (Hyclone, Logan, UT). All cell lines were cultured at 37°C in a humidified atmosphere of 5% CO_2_.

### Patients and tissue specimens

A total of 37 primary NPC patients treated at the Nanfang Hospital, Southern Medical University from 2006 to 2007 were enrolled to immunohitochemical study, including 26 males and 11 females with a median age of 49 years (range, 24-82 years). Meantime, 17 samples out of these 37 patients and another 7 chronic nasopharyngitis tissue samples were get freshly from the Nanfang Hospital, Southern Medical University and frozen in liquid nitrogen until process for RT-PCR examination. All patients were not pretreated with radiotherapy or chemotherapy prior to surgery. For the use of these clinical materials for research purposes, prior consent of the patients and approval from the Ethics Committee of Southern Medical University were obtained. All specimens were confirmed by pathological examination and staging was performed according to the 1992 Fuzhou NPC staging system of China[12,13].

### Extraction of total RNA and RT-PCR

Total RNA was extracted from tissues and cell lines with TRIzol (Invitrogen, Carlsbad, CA) according to the user manual. cDNA was prepared from total RNA by using a First Strand cDNA Synthesis kit (Roche, Indianapolis, IN). Cripto-1 mRNA expressions were determined in 7 cell lines by real-time RT-PCR and in 17 NPC tissue samples, 7 chronic nasopharyngitis tissue samples by RT-PCR analysis.

Expressions of Cripto-1 mRNA in NPC cell lines were detected compared to that in NP69 cell line. For real-time RT-PCR, each reaction was done on an MX3000P instrument (Stratagene, Cedar Creek, TX) with the SYBR^® ^Premix Ex Taq™ kit (Takara bio, Otsu, Japan) in a 25 μl reaction system with 1 μg cDNA following the manufacturer's protocol. ACTB (β-actin) was used as an internal control, and measurements between samples were compared by the threshold cycle of amplification (C_T_). The fold change in expression levels was determined by a comparative C_T _method using the formula: 2^-ΔΔCT^(ΔΔC_T _= (C_T(Cripto-1) _- C_T(β-actin)_)_cancer _- (C_T(Cripto-1) _- C_T(β-actin)_)_NP69_). Primer sequences used for Cripto-1 are: forward 5'- GATACAGCACAGTAAGGAGC -3' and reverse 5'- TAGTTCTGGAGTCCTGGAAG -3'; for β-actin: forward 5'- CACCCAGCACAATGAAGAT -3' and reverse 5'- CAAATAAAGCCATGCCAAT -3'. The primers were designed between different exons and encompassing large introns to avoid any amplification of genomic DNA. QPCR was performed for pre-denaturing at 95°C for 60 seconds, followed by 45 cycles (95°C for 15 seconds, 60°C for 60 seconds and 72°C for 30 seconds). Specificity of amplification products was confirmed by melting curve analysis. All reactions were repeated three times, and the mean fold changes and standard deviation are reported. For RT-PCR reactions, the thermal cycle was defined at 94°C for 5 min, followed by 40 cycles of denaturing at 94°C for 30 s, annealing at 60°C for 60 s and extension at 72°C for 30 s, and a final extension at 72°C for 10 min. PCR products were electrophoresed in 1.5% agarose gels and visualized by ethidium bromide staining to check for nonspecific amplification.

### Western-blot analysis

Cells were washed twice with cold PBS and lysed on ice in RIPA buffer (1 × PBS, 1% NP40, 0.1% SDS, 5 mM EDTA, 0.5% sodium deoxycholate, and 1 mM sodium orthovanadate) with protease inhibitors PMSF (Sangon, Shanghai, China). After concentration measured by the BCA method, equal amounts of protein were electrophoresed on 12% SDS/polyacrylamide gels and subsequently transferred to a polyvinylidene difluoride membranes (PVDF) (Millipore, Billerica, MA) by electroblotting. After blocking for 1 h in Tris buffered saline (pH 7.6, containing 0.1% Tween and 5% non-fat milk) at room temperature, membranes were incubated overnight at 4°C with primary mouse monoclonal antibody against Cripto-1 (R&D Systems, Minneapolis, MN, USA, at 1:1000 dilution), and β-actin (Abcam, Cambridge, UK, at 1:2000 dilution) with gentle shaking. After washing, the membrane was then probed with the appropriate secondary antibody for 60 min at room temperature. Protein binding on the membrane was detected by the enhanced chemiluminescence (ECL) detection system (Pierce, Rockford, IL) according to the manufacturer's instructions. Then band intensity was measured by densitometry using the Quantity One software (Bio-Rad, Hercules, CA). The protein levels were normalized with respect to β-actin protein level.

### Immunohistochemistry analysis

Sections (4 μm thick) of formalin fixed, paraffin wax blocks were cut onto polylysine- coated microscope slides. According to the specification of IHC S-P detection kit (Maixin, Fujian, China): after deparaffinisation in xylene and hydration through graded alcohol, sections were washed and then exposed with ready-to-use proteinase K solution (DakoCytomation, Carpinteria, CA) for 6 minutes at room temperature to enhance antigenicity. Endogenous peroxidase was blocked with 3% hydrogen peroxide for 10 min and non-specific binding was blocked with 5% normal goat serum in phosphate buffered saline for 15 min. Then sections were incubated with first antibody (mouse-anti-human Cripto-1 monoclonal antibody, R&D Systems, Minneapolis, MN, USA) at a concentration of 1: 100 at 4°C overnight. Biotinylated antimouse IgG antibody (Boshide, Wuhan, China) was added for 15 min at 37°C, following the incubation with streptavidin-biotin/horseradish peroxidase complex for 10 min at 37°C. Finally, sections were colored with 3,3'-diaminobenzidine tetrahydrochloride (DAB) for 5 min, lightly counterstained with hematoxylin and mounted. Sections immunostained with PBS replacing primary antibody are used as negative control. A positive control was included with each batch of staining to ensure consistency between consecutive runs.

### Evaluation of staining

The brown-yellow staining of the cytoplasm and the cytoplasmic membrane was considered positive. For each case, the entire stained tissue section was scanned, 5 visual fields at 400× magnification were randomly chosen and 100 cells in each field were counted. The degree of immunointensity was quantified by using the total immunostaining score calculated as the sum of the percent positivity of stained tumour cells and the staining intensity. The percent positivity was scored as '0' (<5%, negative), '1' (5-25%, sporadic), '2' (26-50%, focal), '3' (>50%, diffuse). The staining intensity was score as '0' (no staining), '1' (weakly stained), '2' (moderately stained), and '3' (strongly stained). Cases with weighted scores of less than 3 were defined as negative; otherwise they were defined as positive[14]. No folding, and edging-effect fields were chosen during calculation of 100 cells per five fields. The score assessment was performed independently by two pathologists blinded to the clinical parameters.

### Construction of shRNA expressing vectors

The specific siRNA targeting sequence (5'-AATGACTCTGAATTAAAG-3')[15] is homologous to nt 190-208 of the Cripto-1 mRNA(Gene Bank Accession No. NM_003312). Short hairpin RNA (shRNA) was synthesized and cloned into the pLVTHM vector, which contained H1 promoter and a reporter gene green fluorescent protein (GFP). An invalid RNAi sequence (5'-GCAGGAGCTATGCTACCATCA-3') was used as negative control. The correct insertion of the specific shRNA was further confirmed by sequencing.

### Treatment of NPC cells with shRNA-encoding expression construct

The Cripto-1-specific shRNA-encoding expression construct (pLVTHM-shCripto-1), psPAX2 and pMD2.G were cotransfected to 293FT cell line using the lipofectamine 2000 (Invitrogen, Carlsbad, CA) to produce lentivirus stock, with negative construct as negative control. The titration of lentiviral vectors were determined as described by Tiscornia et al[16]. Briefly, the lentiviral preparation was diluted tenfold serially in PBS (from undiluted to a dilution of 10^-5^). 0.5 × 10^5 ^293FT cells were seeded in each well of the 24-well plate, in a final volume of 500 μl per well. Added 20 μl of each viral dilution to the cells, mixed thoroughly but gently and incubated the cells at 37°C for 48 h. Cells were washed twice with PBS to eliminate leftover virus in the medium. The percentage of labeled cells was determined by FACS. Biological titer (BT = TU/ml, transducing units) was calculated according to the following formula: TU/μl = (P × N/100 × V) ×1/DF, where P = % GFP^+ ^cells, N = number of cells at time of transduction, V = volume of dilution added to each well = 20 μl and DF = dilution factor.

After the titer was determined, the lentivirus stock was transduced to NPC cells according to the manufacture recommendations of BLOCK-iT™ Lentiviral RNAi Expression System (Invitrogen, Carlsbad, CA). Cell lines with stable Cripto-1 knock-down and the negative controls were established by FACS selection for GFP expression and named as CNE-2/GFP^+^/Cripto-1^-^, CNE-2/GFP^+^/mock, C666-1/GFP^+^/Cripto-1^- ^and C666-1/GFP^+^/mock, respectively.

### In vitro cell growth assay

MTT (3-(4, 5-dimethylthiazol-2-yl)-2, 5-diphenyltetrazolium bromide) (Sigma, St. Louis, MO, USA.) cell viability assay, routine checks of the cell growth was performed to assess the proliferation of the transduced cells. The cells were incubated in 96-well microtiter plates at a density of 10^3 ^cells per well and cultured for 1-7 days. Each subsequent day, 20 μl of 5 mg/mL MTT were added to each well. After being incubated for 4 h at 37°C, the supernatants were removed carefully. 150 μl of dimethyl sulfoxide (Sigma, St. Louis, MO) were added to each well and thoroughly mixed for 10 minutes. The absorbance value (OD) of each well was measured with a microplate reader (BioRad, Hercules, CA) set at 490 nm. All experiments were performed in triplicate.

### FACS analysis

Cell cycle distribution was analyzed by EPICS ACTRA FACScan system (Beckman Coulter, US). 1 × 10^6 ^cells were harvested, washed with ice-cold PBS, fixed by ice-cold 70% ethanol at 4°C overnight and labeled with DNAcon3 flow cytometry staining kit (Consults, Italy) according to the manufacturer's instructions. All assays were performed in triplicate, and the results were analyzed by Mod-fit software (Becton-Dickson, Franklin Lakes, NJ, USA)

### Tumour cell invasion assay

We used modifed Boyden chambers with filter inserts (pore size, 8 μm) coated with Matrigel (Becton Dickinson Labware) in 24-well dishes. Tumour cells in serum-free medium (300 μl containing 1 × 10^5 ^cells) were added to the top chamber. The bottom chamber was prepared with 10% FBS as a chemoattractant. After 48 h incubation, the noninvasive cells were removed with a cotton swab. The cells that had migrated through the membrane and had stuck to the lower surface of the membrane were fixed with methanol for 15 minutes and stained with haematoxylin. For quantification, the cells were counted under a microscope in 5 predetermined fields at ×200.

### In vivo experiments

The experimental protocol was approved by the Animal Care and Use Committee of Nanfang Medical University. A total of 5 mice, 4-5 weeks old and 18-20 g in weight, were provided by the Central Animal Facility of Nanfang Medical University and were bred in a specific pathogen-free condition. All the 5 mice underwent subcutaneous injection of 100 μl cell suspension of CNE-2 (1 × 10^6^) in the up axillary dorsal scapula region as control group, and CNE-2/GFP^+^/Cripto-1^- ^(1 × 10^6^) in the low inguinal region. Tumor diameters were measured three times per week with a caliper, and the volumes of tumors were calculated by the following formula: *a *× *b*^2^/2, where *a *is the largest diameter of the tumor and *b *is the shortest diameter. GFP fluorescence images of the dopey mice were got by an *in vivo *fluorescence instrument. All mice were sacrificed 3 weeks after innoculation and tumors were harvested from mice and H&E (hematoxylin and eosin) routine stains were executed. To examine the proliferative activity of xenotransplant tumor after Cripto-1 gene silencing, immunohistochemistry was performed to detect the expression of Ki67 (rabbit-anti-human Ki67 polyclonal antibody, Boshide, Wuhan, China) in tumor tissues and the percentages of tumor cells immunolabeled for Ki67 were calculated. Approximately 1,000 cells were counted in each section. Results are presented as means of 5 counts.

### Statistical analysis

Quantitative values were expressed as means ± SD. All statistical analyses were performed in the SPSS 15.0 statistical software package. Fisher's exact test was used to analyze the relationship between Cripto-1 expression and clinicopathologic characteristics. Tumor cell invasion assay, MTT assay and FACS analysis were tested by ANOVA, with LSD test for multiple comparisons. Independent-samples *t- *test was used to compare the values of the test and control samples *in vivo *experiment. A *p *value less than 0.05 was considered statistically significant.

## Results

### Expression analysis of Cripto-1 by real-time PCR, RT-PCR and western blot

**Figure 1:**
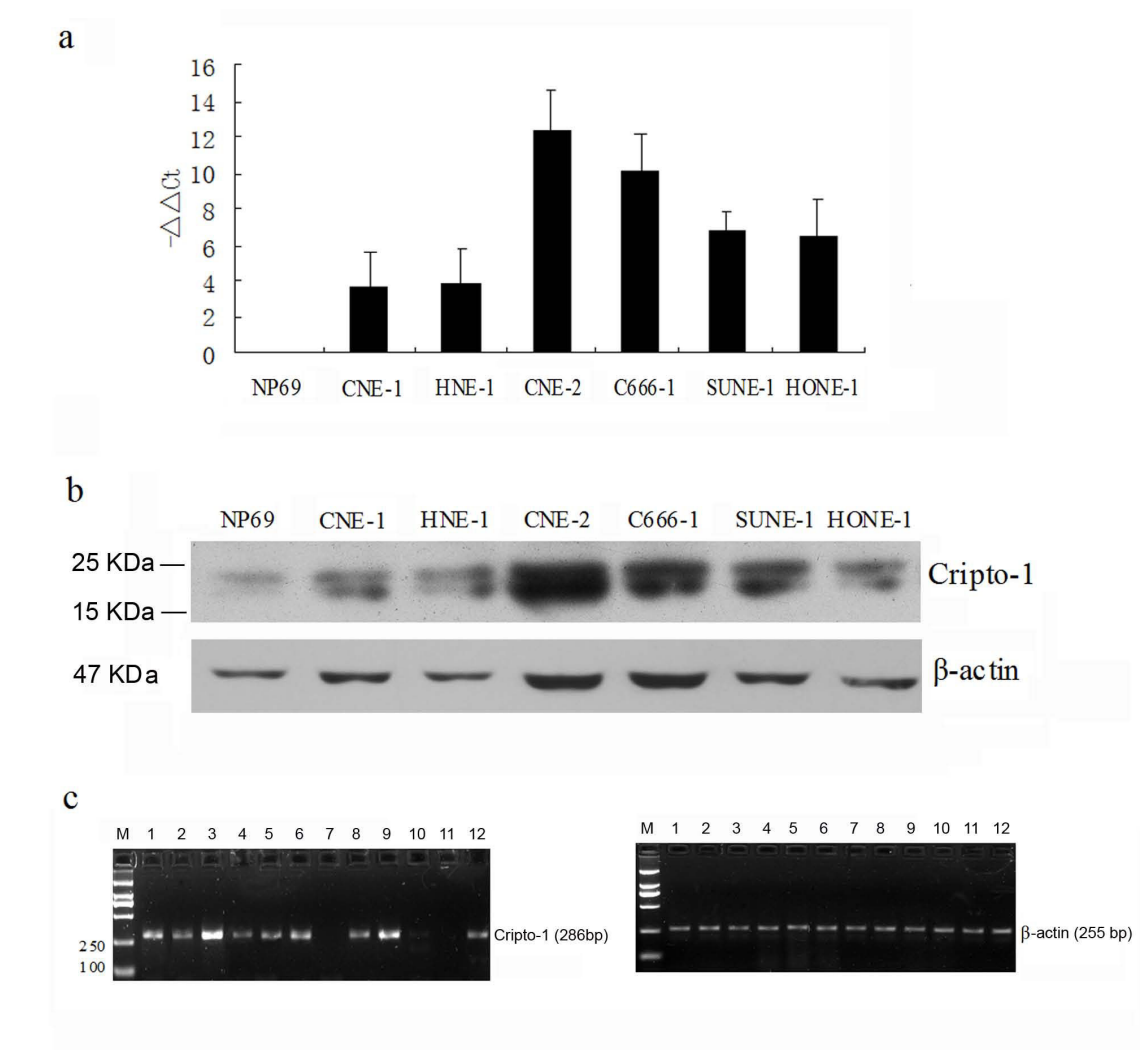
**Summary of Cripto-1 expressions in NPC cell lines and tissues**. Expressions of Cripto-1 mRNA and protein in immortalized nasopharyngeal epithelial cell line (NP69), and NPC cell lines (SUNE-1, C666-1, HNE-1, HONE-1, CNE-1 and CNE-2) were examined by real-time RT-PCR **(a) **and Western blotting **(b)**. Compared to NP69, Cripto-1 expressions were up-regulated in all NPC cell lines (*p *< 0.05, respectively). **(c) **1.5% agarose electrophoresis of Cripto-1 RT-PCR products in NPC tissues. Representative results from 12 NPC cases are shown. β-actin was used as an internal quantitative control. M: Marker; 1~6, 8, 9, 12: positive expression of Cripto-1 mRNA; 7, 10, 11: negative expression of Cripto-1 mRNA.

To determine correlation of Cripto-1 with NPC, semi-quantitative real-time PCR and western blot analysis were performed to evaluate the expression levels of Cripto-1 transcripts and protein in an immortalized primary nasopharyngeal epithelial cell line (NP69) and 6 NPC cell lines (SUNE1, C666-1, CNE-2, CNE-1, HONE-1 and HNE-1). As shown in Figure 1a and 1b, compared with NP69 cell, the expression levels of Cripto-1 mRNA (p < 0.01) and protein (p < 0.05) are significantly up-regulated in all 6 NPC cell lines (Detailed qRT-PCR data are shown in additional file 1). Cripto-1 mRNA was highly expressed in CNE-2 cell line with high metastatic ability, lowly expressed in CNE-1 cell line with no metastatic ability, and moderately in C666-1, SUNE-1, HONE-1 and HNE-1, which indicated that CNE-2 cell line is a suitable cell model for RNAi targeting Cripto-1 mRNA. In addition, we included C666-1, which remains latently infected by EBV in our study to better representative of NPC tumors. The expression levels of Cripto-1 mRNA were also examined in 17 NPC tissue samples, 7 chronic nasopharyngitis tissue samples by semi-quantitative RT-PCR analysis. Cripto-1 was higher expressed in 13 of 17 (76.5%) carcinomatous tissues compared with 2 of 7 (28.6%) control inflammatory tissues (Table 1, *p *= 0.042) (Figure 1c).

**Table 1:** mRNA Expression of Cripto-1 detected by RT-PCR in 17 NPC and 7 chronic nasopharyngitis tissue samples

	mRNA expression					
			
Sample	positive		negative		Total	*p *value
			
	n	%	n	%		
**NPC**	13	76.5	4	23.5	17	**0.042**
**chronic nasopharyngitis**	2	28.6	5	71.4	7	

### Cripto-1 was overexpressed in NPC tissues

**Figure 2:**
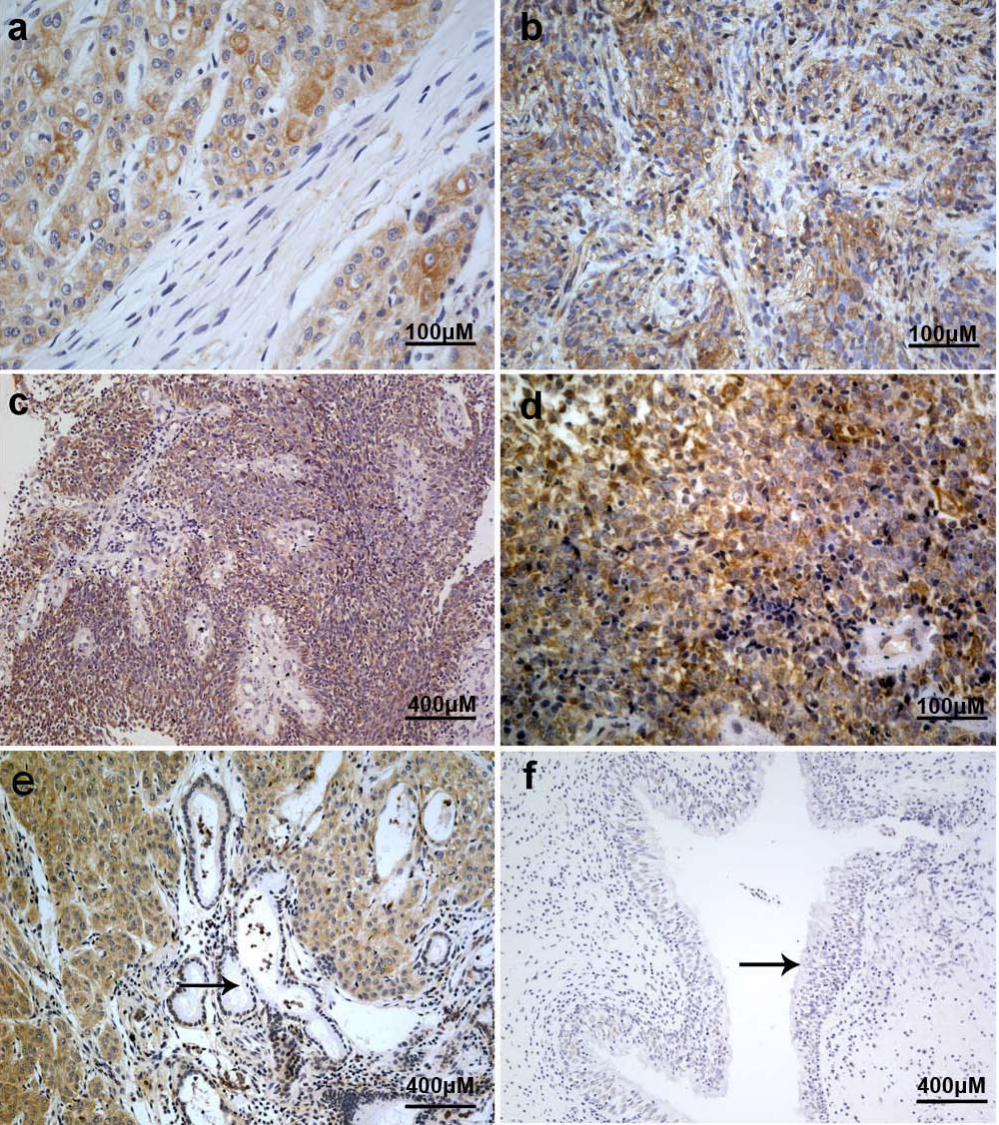
**Representative pictures of Cripto-1 expression evaluated by immunohistochemical staining in human NPC samples**. **(a) **and **(b) **Positive expression of Cripto-1 in WHO type I and III NPC samples (Original magnification ×400). **(c) **and **(d) **Positive expression of Cripto-1 in WHO type II NPC samples (Original magnification, ×100 and ×400, respectively). **(e) **and **(f) **Negative Cripto-1 staining of residual gland and normal nasopharyngeal epithelial tissue (arrow: residual gland and normal epithelial cells; Original magnification, ×100).

To investigate whether Cripto-1 abnormalities are linked to human NPC, we analyzed Cripto-1 protein expression in 37 paraffin-embedded, archival NPC tissues. Cripto-1 protein was detected in 20 (54.1%) cases. Cripto-1 staining was mostly observed in the cytoplasm and cytoplasmic membrane of carcinoma cells (Figure 2). No specific Cripto-1 staining was observed in normal adjacent nasopharyngeal epithelial cells and stroma cells in surrounding tissues.

### Relationship between clinicopathological features and Cripto-1 expression in NPC

The relationship between clinicopathological features and Cripto-1 expression in NPC is summarized in Table 2. Interestingly, we observed that Cripto-1 expression was positively correlated with N classification (*p*= 0.034), distant metastasis (M classification, *p *= 0.036) and clinical stage (*p *= 0.007) of NPC patients. With the evolvement of NM stage and clinical stage, the positive rate of Cripto-1 expression in NPC tissues increased.

**Table 2:** Correlation between the clinicopathological features and expression of Cripto-1

		Cripto-1		
			
Clinicopathological variables	Cases (n = 37)	positive (%)	negative (%)	*p *value
			
		n = 20	n = 17	
**Gender**				0.627
Male	26	14 (53.8)	12 (46.2)	
Female	11	6 (54.5)	5 (45.5)	
**Age(years)^a^**				0.540
≤ 49	21	11(52.4)	10 (47. 6)	
>49	16	9 (56.3)	7 (43.7)	
**Pathological classification(WHO)**				0.062
Type I-II	17	12(70.6)	5 (29.4)	
Type III	20	8 (40.0)	12 (60.0)	
**Depth of invasion** T classification				0.474
T1-2	27	14(51.9)	13 (48.1)	
T3-4	10	6(60)	4(40)	
**Lymph node metastasis** N classification				0.034^c^
N_0_	9	2 (22.2)	7 (77.8)	
N_1-3_	28	18(64.3)	10 (35.7)	
**Distant metastasis**				0.036^c^
Yes	5	5(100.0)	0(0.0)	
No	32	15 (48.4)	17(51.6)	
**Clinical Staging^b^**				0.007^c^
I-II	15	4 (26.7)	11 (73.3)	
III-IV	22	16(72.7)	6(27.3)	

^a^grouping of age was performed according to median.

^b^clinical staging was performed according to 1992 Fuzhou NPC staging system of China.

^c ^statistical significance (*p *< 0.05)

### Lentivirus-mediated RNAi silencing inhibited the expression of Cripto-1 mRNA and protein in CNE-2 and C666-1 cell lines

**Figure 3:**
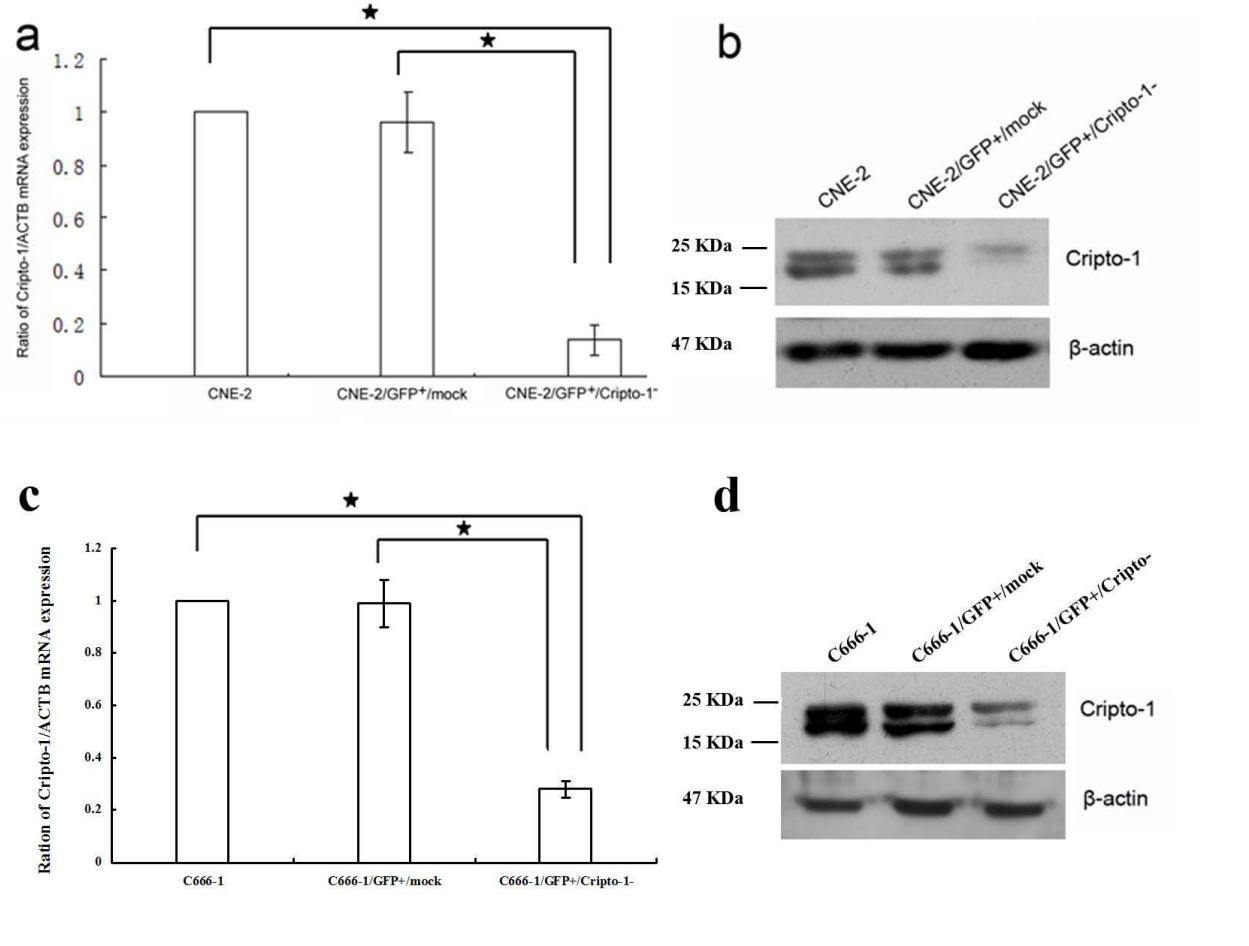
**Summary of Cripto-1 expression before and after lentivirus-mediated RNA interference**. Real-time RT-PCR showed that the Cripto-1 mRNA in CNE-2 **(a) **and C666-1 cells **(c) **were effectively knockdown by RNAi. * *p *< 0.05. Protein expression were detected by western blot analysis, Cripto-1 were specifically inhibited in CNE-2/GFP^+^/Cripto-1^- ^**(b) **and C666-1/GFP^+^/Cripto-1^- ^cells **(d)**. (*p *< 0.05, respectively).

To investigate whether Cripto-1 overexpression involves in cell growth, cell cycle progression and invasive activity, the CNE-2 and C666-1 cell lines were transduced with recombinant lentivirus of small interference RNA targeting Cripto-1 and the negative control lentivirus (GFP^+^/mock-lentivirus). After a selection of GFP expression by FACS, we got CNE-2/GFP^+^/Cripto-1^- ^and CNE-2/GFP^+^/mock, C666-1/GFP^+^/Cripto-1^- ^and C666-1/GFP^+^/mock sublines, with high percentages (more than 95%) of transductants expressed GFP, indicating a high and stable transduction of lentiviral vector system. As shown in Figure 3, the results of quantitative RT-PCR and western blot assays revealed that the expression of Cripto-1 in CNE-2/GFP^+^/Cripto-1^- ^and C666-1/GFP^+^/Cripto-1^- ^were markedly decreased compared to that in parent cells and negative controls (*p *< 0.05 respectively), which demonstrated that RNAi technique was an effective way to modulate the Cripto-1 expression in NPC cell lines.

### Suppression of Cripto-1 expression decreased cell growth

**Figure 4:**
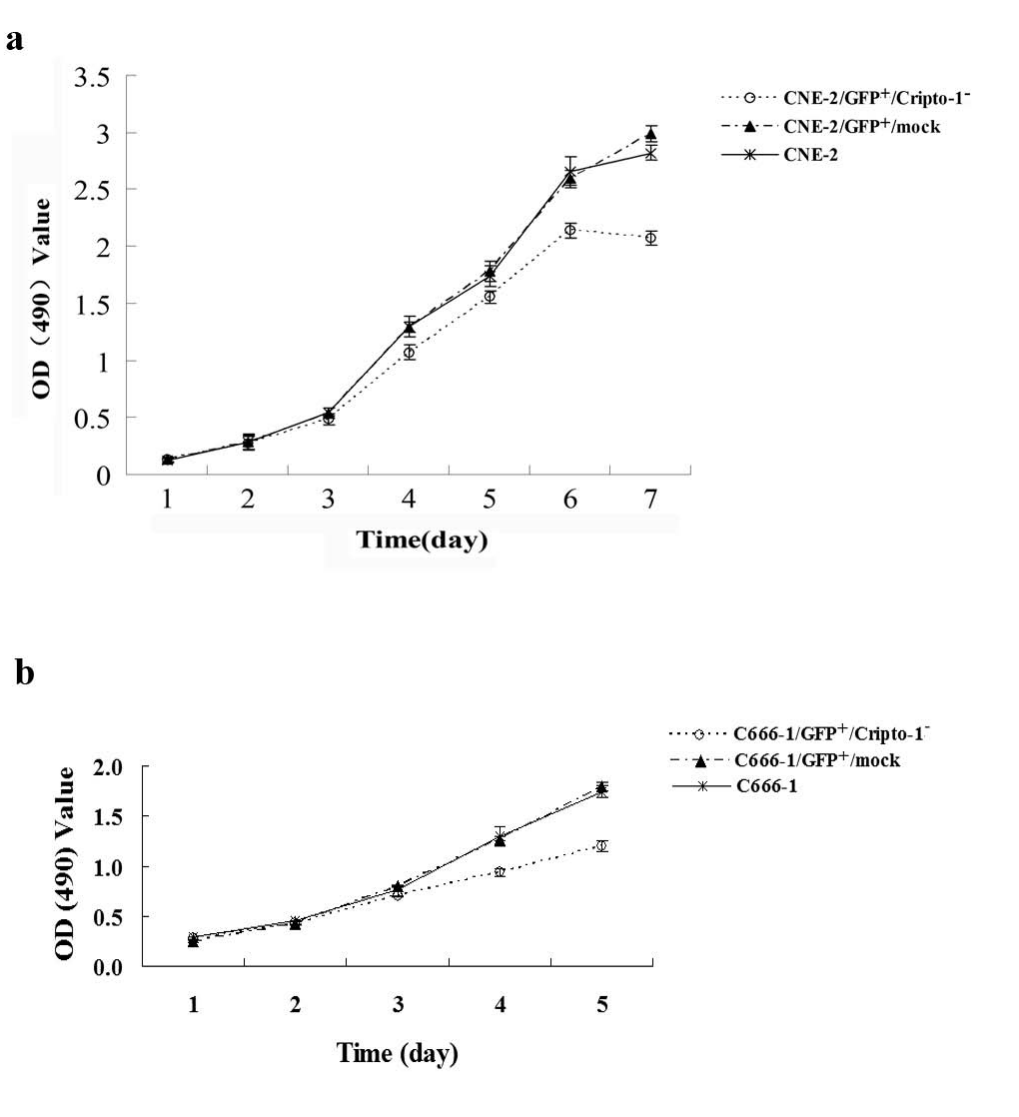
**Growth curves of CNE-2 and C666-1 cell lines treated with lentivirus mediated RNAi**. The cell growths were assessed by MTT method. Each value represents the mean ± SD of absorbance value (OD) for cells. CNE-2/GFP^+^/Cripto-1^- ^cells **(a) **and C666-1/GFP^+^/Cripto-1^- ^cells **(b) **exhibited a slower growth rate than that of the control cells. (*p *< 0.01, respectively).

As shown in Figure 4, after Cripto-1-shRNA-lentivirus transduction, the growth of CNE-2/GFP^+^/Cripto-1^- ^(*F *= 32.364, *P *< 0.01) and C666-1/GFP^+^/Cripto-1^- ^cells(*F *= 44.772, *P *< 0.01) were evidently decreased. However, the proliferation of control cells (CNE-2/GFP^+^/mock and C666-1/GFP^+^/mock cells) showed no significant alteration compared to parent cells during the time course. These time-effect curves indicated that Cripto-1 knock-down could inhibit the growth of NPC cells *in vitro*.

### Cripto-1 knock-down wasn't involved in the regulation of cell cycle progression

To explore the possible underlying mechanisms of Cripto-1 suppression in inhibiting CNE-2 cell growth, the effect of Cripto-1 expression on cell cycle was analyzed by flow cytometry. As shown in Table 3, no significant effect on the cell cycle of CNE-2 cells was observed after Cripto-1 knock-down.

**Table 3:** Percentage of G1, S, G2 stage cells of CNE-2, CNE-2/GFP+/mock and CNE-2/GFP^+^/Cripto-1^- ^cells

Cell	G1(%)	S(%)	G2(%)
CNE-2	53.95 ± 0.5	34.85 ± 2.39	11.25 ± 2.78
CNE-2/GFP^+^/MOCK	56.85 ± 3.7	34.175 ± 2.58	9.025 ± 1.79
CNE-2/GFP^+^/Cripto-1^-^	54.925 ± 4.94	34.25 ± 5.08	10.8 ± 2.11
*p value*	0.531	0.932	0.38

### Cripto-1 knock-down inhibited invasion of human NPC cells

**Figure 5:**
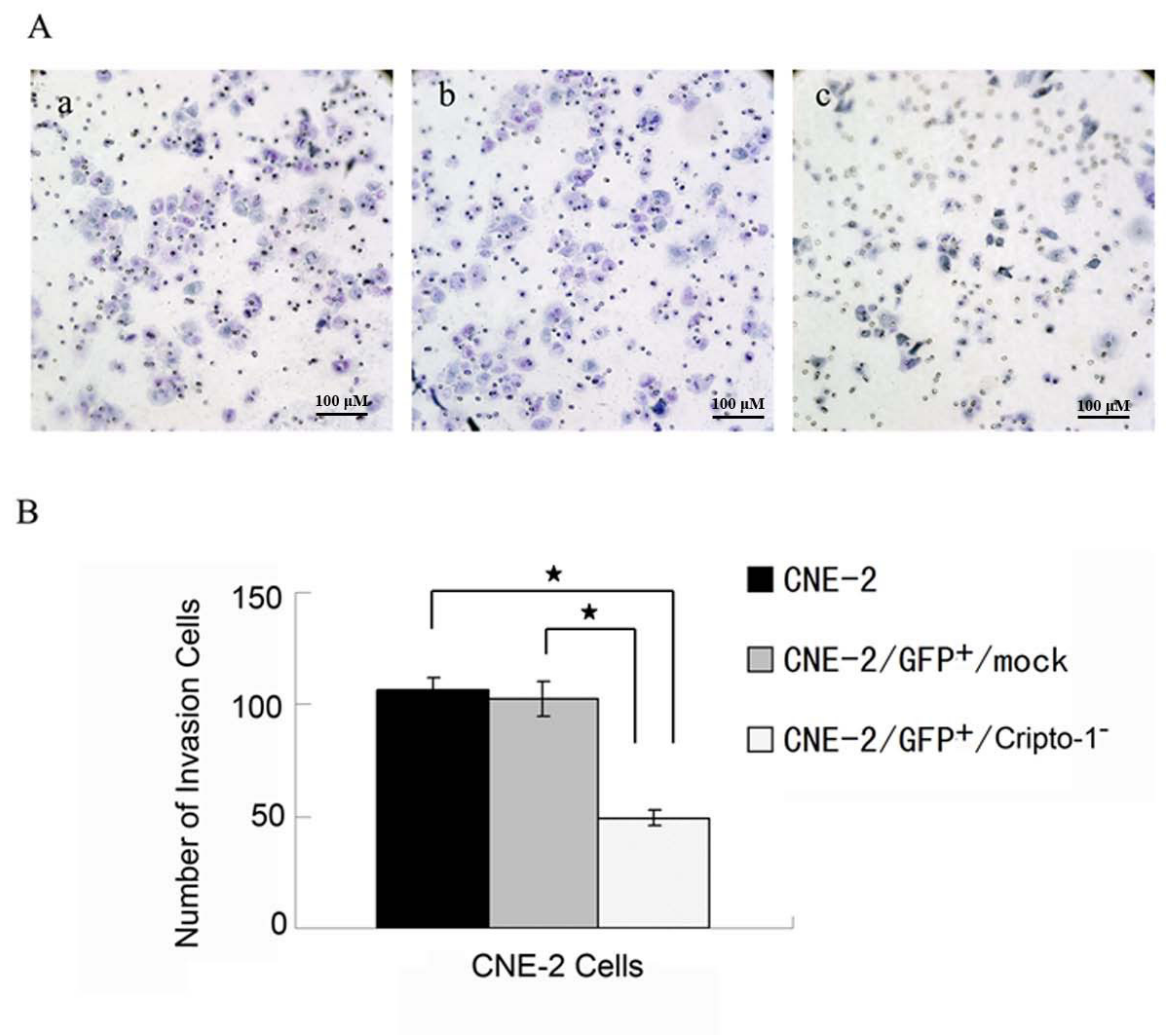
**Cripto-1 knock-down inhibited the invasion of CNE-2 cell lines**. Effects of Cripto-1 knockdown on cell invasive potency were determined by an *in vitro *invasion assay. (**A**) 48 hr after seeded, the cells on the upper chambers were removed, and the invasive cells on the lower surface of the membrane were fixed and stained with hematoxylin, and visualized at ×200 magnification. a: CNE-2, b: CNE-2/GFP^+^/mock, c: CNE-2/GFP^+^/Cripto-1^-^. (**B**) The cells on the lower surface of the membrane were counted in five randomly selected fields. Each value represents the average of triplicate determinations expressed as the mean ± SD. (* *p *< 0.01).

On the other hand, we performed an invasion assay in a modifed Boyden chamber to examine the effect of Cripto-1 knock-down on the invasive potency of the CNE -2 cells *in vitro*. Movement of cells through Matrigel-coated Boyden chambers mimics the early steps of tumor invasion. After cultivation for 48 h, the mean ± SD of cells attached to the lower surface of the membrane of CNE-2-treated different groups, as indicated previously, were as follows: parent CNE-2, 106.4 ± 5.86; CNE-2/GFP^+^/mock, 102.6 ± 7.4; and CNE-2/GFP^+^/Cripto-1^-^, 49.2 ± 3.27. Cripto-1 knock-down cells exhibited significantly reduced invasive tendencies compared with the parent CNE-2 cells and the CNE-2/GFP^+^/mock cells (F = 153.754, *P *< 0.01; Figure 5). Therefore, disruption of endogenous Cripto-1 expression resulted in inhibition of cell invasion in NPC cells.

### Cripto-1 gene silencing suppressed proliferation of CNE-2 cells in vivo

**Figure 6:**
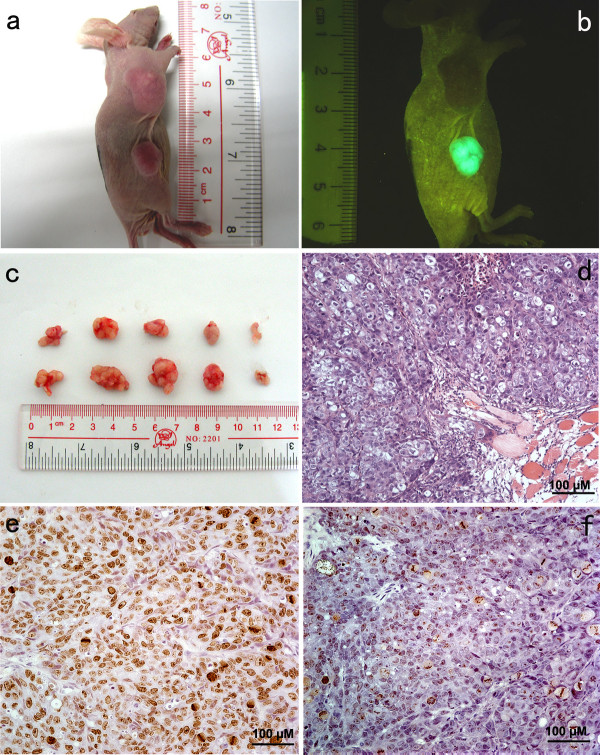
**Cripto-1 gene silencing suppresses cell proliferation *in vivo***. **(a) **representative mouse bearing tumors (up was in CNE-2 control group, down was in CNE-2/GFP^+^/Cripto-1^- ^group). (**b) **external whole-body fluorescence images of the same mouse. (**c) **the external images of xenotransplant tumors (top was in CNE-2/GFP^+^/Cripto-1^- ^group, bottom was in CNE-2 control group). (**d) **H&E stains of xenotransplant tumors, (×400). **(e) **expression of Ki67 in xenotransplant tumors of CNE-2 control group (×400). **(f) **expression of Ki67 in xenotransplant tumors of CNE-2/GFP^+^/Cripto-1^- ^group (×400).

Of the 5 mice that were injected subcutaneously with 1 × 10^6 ^CNE-2 and CNE-2/GFP^+^/Cripto-1^- ^cells in up axillary dorsal scapula region and low inguinal region respectively, all of them developed evident tumors at the end of this experiment. However, the mice treated with CNE-2/GFP^+^/Cripto-1^- ^cells showed that the growth of tumor was significantly suppressed compared with those treated with CNE-2 cells (Figure 6a, b and 6c). Three weeks after inoculation, the average tumor volume (0.410 ± 0.17 cm^3^) in later group was significantly lower (*p *= 0.044) than that (1.153 ± 0.67 cm^3^) in the former group. The average tumor weight (0.51 ± 0.2 g) was lower (*p *= 0.026) than that in control group (1.49 ± 0.7 g) too. No obvious difference was found in body weight of mice in the treated and control groups (data not shown). H&E stains showed that all the tumor tissues were low-differentiation cell carcinoma (Figure 6d). Additionally, immunohistochemistry method exhibited that there was a significant difference (*p *< 0.01) in Ki67 index between the RNAi group (49.2 ± 4.8%) and the control group (89.6 ± 3.4%) (Figure 6e and 6f). These data indicate that Cripto-1 gene silencing can suppress the proliferation of CNE-2 cells in this experimental condition.

## Discussion

Human Cripto-1 is increased in several types of cancer and can function as an oncogene *in vitro *and *in vivo*[9,17,18]. However, there has been no report about the relations between Cripto-1 and NPC. In this study, we presented the first evidence that Cripto-1 was overexpressed at both transcriptional and translational levels in NPC cell lines. Using immunochemistry and semi-quantitative RT-PCR analysis, we found that Cripto-1 protein was expressed in 54.1% (20 of 37 cases) and 76.5% (13 of 17 cases) human NPC tissues, respectively. However, only 28.6% (2 of 7 cases) nasopharygitis showed Cripto-1 positive. We also found that Cripto-1 overexpression was significantly associated with N classification, distant metastasis, and clinical stage. Our results highlight the clinical significance of Cripto-1 in NPC. Expression of Cripto-1 shows 50-80% variance, as detected by immunohistochemistry, in different types of tumour tissues. More recently, Cripto-1 was detected in almost half of the breast cancer tissue samples analyzed and found to correlate with advanced stage disease[19]. Our results are consistent with these reports and thus, NPC joins the list of the tumors that express Cripto-1.

Until now, numerous *in vitro *and *in vivo *studies have shown that cripto-1 act as an important factor during tumorigenesis by promoting cell proliferation, survival, migration and invasion. Overexpression of Cripto-1 cDNA in normal mouse fibroblasts induce these cells to grow in soft agar and increase growth rates in several human breast cancer cell lines[20]. Human MCF-7 breast cancer cells that overexpress Cripto-1 proliferate at higher rates in serum-free medium, form increased numbers of colonies in soft agar, are more resistant to apoptosis when grown under anchorage independent conditions, and show increased propensity to invade and migrate *in vitro*[21]. MMTV-Cripto-1 transgenic mice and WAP-Cripto-1 transgenic mice that overexpress the human Cripto-1 transgene showed increased incidence of mammary gland tumors[9,18]. To explore the possible role of Cripto-1 in promoting NPC cell proliferation and invasion, we knocked down the expression of Cripto-1 in CNE-2 and C666-1 by lentivirus-mediated RNAi silencing. Then through a continuous MTT assay, we found that the cell growth was suppressed after the inhibition of endogenous Cripto-1 protein, suggesting a role of Cripto-1 in promoting tumor cell growth. However, flow cytometry analysis showed no significant change on the cell cycle of CNE-2 cells after Cripto-1 silence. This result suggests that Cripto-1 might promote cell growth in other pathway rather than impact on cell cycle. However, due to high proliferative rate of CNE-2 cells and incomplete RNAi suppressive effect (knock-down rather than knock-out), the negative results of cell cycle analysis need to be further verified.

To investigate the proliferation of the tumor cells *in vivo *after Cripto -1 gene silencing, we employed the xenotransplant nude mice model with whole-body visualizing instrument. Data showed that both the average volume and weight of tumor in CNE-2/GFP^+^/Cripto-1^- ^xenotransplant mice group were significantly lower than those in the control group, indicating that the Cripto-1 gene silencing could partially inhibit the growth of CNE-2 cells *in vivo*. Ki67, a nuclear protein regulating cell cycle, is a biomarker of cell proliferation. Our results showed that the number of cells immunolabeled for Ki67 in the CNE-2/GFP^+^/Cripto-1^- ^xenotransplant tumors was significantly less than that in the control group, suggesting that a decreased proliferation of CNE-2 cells could be achieved by Cripto-1 knock-down.

Association of Cripto-1 with metastatic potential has been found in a few human cancers. Ciardiello et al. have reported that Cripto-1 mRNA expression is found in 68.2% (30/44) of primary colonic cancers and 61.8% (21/34) of liver metastases, but in only 1.5% (1/65) of normal tissues[22]. Furthermore, the Cripto-1 expression level was increased in lymph node metastases compared with their primary tumours[23]. In our study, Cripto-1 mRNA was highly expressed in CNE-2 and C666-1 cell lines which are highly metastatic and proliferative. In the contrary, in CNE-1 cell line with no metastatic ability, Cripto-1 was expressed lowly. We also found Cripto-1 expression correlates with metastasis in NPC patients. In addition, by *in vitro *invasion assay, substantial suppression of cell invasion was observed after endogenous Cripto-1 interference. All of these data suggest an association between Cripto-1 and tumour cell motility, invasion, and metastasis. Thus, our studies have shown that Cripto-1, as a potentially oncogenic protein, might play an important role in the tumorigenesis and progression of NPC.

However, the possible mechanism and the exact mode of Cripto-1 action during tumor metastasis and progression are still largely unknown. Although, we have not demonstrated clearly the mechanisms underlined the inhibition of Cripto-1 gene silencing, it has been evidenced that in addition to functioning as a Nodal co-receptor, Cripto-1 has been shown to mediate signaling of other TGF-*β *ligands, such as Activin and Xenopus Vg1 and its ortholog in mouse GDF1[24]. In contrast, binding of Cripto-1 to Activin and TGF-*β*1 can inhibit Activin and TGF*β*-1 signaling in mammalian cells[25]. Moreover, Cripto-1 can also activate the ras/raf/MAPK and PI3-K/AKT/GSK-3*β *intracellular signaling pathways independently of Nodal and ALK4[26,27]. Some data suggest that Cripto-1 may be involved in regulating integrin signaling either directly by binding to integrins and subsequently activating integrin signaling or indirectly by regulating the expression of extracellular matrix proteins which are also capable of binding integrins and activating integrin signaling[5]. Several studies have also suggested that the Wnt/b-catenin/Lef-1 signalling pathway may cross-talk with the Cripto-1 signaling pathway, regulating cell adhesion and migration[17,28,29]. Strizzi et al. have reported that Cripto-1 may promote the increased expression of markers and signaling molecules associated with EMT[17]. Also, in the Cripto-1 transgenic mammary gland tumors, the zinc-finger repressor transcription factor, Snail, known to down-regulate or interfere with the normal expression of E-cadherin, was detected at significantly higher levels as compared to normal control mammary tissue, thus suggesting a novel link between Cripto-1 expression and Snail activity[17].

Since high expression of Cripto-1 can be detected in human cancers, as compared to normal tissues, this signaling pathway might represent a target for cancer therapy. This is supported by findings describing the use of antisense oligonucleotides that reduce Cripto-1 expression and cause significant reduction of cell proliferation *in vitro*[30]. In addition, neutralizing antibodies against Cripto-1 were able to significantly inhibit tumor cell growth in two xenograft models with testicular and colon cancer cells that express very high levels of Cripto-1[25]. Moreover, rat monoclonal antibodies directed against the EGF-like domain of the Cripto-1 peptide also produced a significant inhibition of *in vitro *and *in vivo *growth of colon cancer and leukemia cells[30,31]. Our data also demonstrated that lentivector-mediated RNAi was feasible for the inhibition of the growth and invasion of NPC cells *in vitro and in vivo*, indicating the siRNA sequences targeting Cripto-1 could be a potential target for gene therapy of NPC.

## Conclusion

In summary, this study demonstrated that the level of expression of Cripto-1 was significantly increased in NPC. Moreover, Cripto-1 expression correlated evidently with the malignant status of NPC. Finally, we showed that Cripto-1 might play a role in the tumorigenesis and progression of NPC by promoting the growth and invasion of NPC cells both *in vitro *and *in vivo*. These findings provide new insight into understanding the molecular mechanism involved in NPC carcinogenesis and progression, and may lead to the development of new approaches for effective diagnosis and therapy. However, more work is still needed to clarify the mechanism of Cripto-1 in the development and progression of NPC and its related signaling pathway in NPC.

## Competing interests

The authors declare that they have no competing interests.

## Authors' contributions

Wu ZR designed the research. Wu ZR and Li G carried out the molecular genetics studies and data analysis. Li G collected the NPC tissues. Wu LR prepared the tissue slides WENG DS drafted and revised the manuscript. WENG DS and Li XP gave advises on the work and helped in the interpretation of the data. Yao KT supervised all the work. All authors read and approved the final version of the manuscript.

## Pre-publication history

The pre-publication history for this paper can be accessed here:

http://www.biomedcentral.com/1471-2407/9/315/prepub

## Supplementary Material

Additional file 1 **original qRT-PCR data of Cripto-1 in 7 cell lines**. The file provided represent the original real-time RT-PCR data of Cripto-1 in 7 cell lines and 293e cell. Click here for file
