# Supplementary material for: Cripto-1 overexpression is involved in the tumorigenesis of nasopharyngeal carcinoma
Source: BMC Cancer. 2009 Sep 6;9:315. doi: 10.1186/1471-2407-9-315 (PMC2751776; doi:10.1186/1471-2407-9-315)
Supplement: Additional file 1 — original qRT-PCR data of Cripto-1 in 7 cell lines. The file provided represent the original real-time RT-PCR data of Cripto-1 in 7 cell lines and 293e cell. [file 1471-2407-9-315-S1.doc]

**Suppl. Table1. primary qRT-PCR data of Cripto-1 in NPC** cell lines

|  | β-actinCt1 | β-actinCt2 | β-actinCt2 | cripto-1Ct1 | cripto-1Ct2 | cripto-1Ct3 |
| --- | --- | --- | --- | --- | --- | --- |
| NP69 | 17.28 | 16.61 | 17.7 | 35.89 | 38.83 | 38.61 |
| CNE1 | 15.79 | 22.04 | 20.69 | 32.65 | 40.54 | 35.81 |
| CNE2 | 26.09 | 26.54 | 25.57 | 34.73 | 34.71 | 35.46 |
| SUNE-1 | 19.48 | 18.56 | 19.17 | 32.1 | 34.13 | 32.15 |
| HNE-1 | 16.31 | 16.33 | 17.12 | 33.34 | 33.66 | 32.93 |
| HONE-1 | 19.46 | 18.67 | 18.3 | 34.11 | 33.22 | 31.42 |
| C666-1 | 23.06 | 23.54 | 22.91 | 34 | 34.52 | 32.29 |
| 293e | 18.76 | 20.25 | 17.91 | N/A | N/A | N/A |
